# On the evolutionary origins of host–microbe associations

**DOI:** 10.1073/pnas.2016487118

**Published:** 2021-02-22

**Authors:** Michael Sieber, Arne Traulsen, Hinrich Schulenburg, Angela E. Douglas

**Affiliations:** ^a^Max Planck Institute for Evolutionary Biology, D-24306 Plön, Germany;; ^b^Zoological Institute, Christian-Albrechts-University Kiel, D-24118 Kiel, Germany;; ^c^Department of Entomology, Cornell University, Ithaca, NY 14853;; ^d^Department of Molecular Biology and Genetics, Cornell University, Ithaca, NY 14853

**Keywords:** host, microbiota symbiosis, microbiota evolution, dispersal

## Abstract

Many microorganisms with high prevalence in host populations are beneficial to the host and maintained by specialized transmission mechanisms. Although microbial promotion of host fitness and specificity of the associations undoubtedly enhance microbial prevalence, it is an open question whether these symbiotic traits are also a prerequisite for the evolutionary origin of prevalent microbial taxa. To address this issue, we investigate how processes without positive microbial effects on host fitness or host choice can influence the prevalence of certain microbes in a host population. Specifically, we develop a theoretical model to assess the conditions under which particular microbes can become enriched in animal hosts even when they are not providing a specific benefit to a particular host. We find increased prevalence of specific microbes in a host when both show some overlap in their lifecycles, and especially when both share dispersal routes across a patchy habitat distribution. Our results emphasize that host enrichment per se is not a reliable indicator of beneficial host–microbe interactions. The resulting increase in time spent associated with a host may nevertheless give rise to new selection conditions, which can favor microbial adaptations toward a host-associated lifestyle, and, thus, it could be the foundation for subsequent evolution of mutually beneficial coevolved symbioses.

## Significance

Animals can provide benefits to their associated microbes—and these can, in turn, positively affect their hosts. But how do such mutually beneficial associations arise in the first place? In particular, when animal and microbe initially have independent lifestyles, this is not clear. By developing a model of animal and microbial life cycles on patchy habitats, we show how their overlapping ecologies of development and dispersal can lead to the enrichment of certain microbes in the dispersing animals, even in the absence of specific mutualistic benefits. This enrichment can then set the stage for the evolution of more specific host–microbe associations, which also implies that host enrichment per se is not an indicator of a beneficial host–microbe symbiosis.

Animals live in a microbial world (1). Microorganisms (bacteria, archaea, and unicellular eukaryotes) evolved and diversified long before the origin of animals (2), and microorganisms inhabit all environments utilized by animals, as well as some habitats that are hostile for animal life (3). In this context, it is unsurprising that microorganisms also colonize animals (1). Only a minority of microbial colonists are pathogens, while many are benign, and some are required for sustained health and reproduction of the animal host. The evolutionary origins of beneficial microorganisms are varied. Some have arisen by amelioration of pathogens, but most are likely to have evolved directly from free-living microorganisms (4–6).

The aim of our study is to assess how such a host-associated lifestyle can evolve in free-living microorganisms, even if the ancestral microorganism initially does not provide a specific benefit for the host, or is even detrimental to its fitness. Our focus is thus distinct from the common assumption that the first evolutionary stage is already characterized by a service provided by the microbial partner which enhances the fitness of the host (7–9). Such a service may be nutritional or provide defense against natural enemies, and it is predicted to generate the selection pressure for host choice of the most favorable microbial taxa. But is this beneficial microbial service necessary for the initial interaction, or can certain microorganisms become enriched in animal hosts without any immediate and specific beneficial effects on the host?

To answer this question, we develop and analyze a theoretical model to investigate the fate of different microorganisms in hosts that neither discriminate between nor exclusively benefit from the microorganisms. We assume that microorganisms can transition between a free-living and a host-associated lifestyle. The microorganisms and hosts cooccur in a landscape of patchily distributed habitats, which is common for many real-world scenarios. Throughout the paper, we use rotting fruits as an explicit reference example for this setup. Rotting fruits provide a substrate for various microorganisms (both bacteria and yeasts) (10), they show a patchy distribution in space and time (11), and they are commonly a resource for invertebrates, including the larvae and adults of many drosophilid fly species. As the insects feed, microorganisms can be taken up from the rotting fruit and accumulate in the insect gut. There is evidence that, in *Drosophila melanogaster*, many persist and even proliferate for varying lengths of time, before being released into the environment via the feces (12–14). Nevertheless, the theoretical model is relevant to a diversity of situations, as elaborated in *Discussion*.

We investigate the conditions that promote overrepresentation of specific microbes in the host, focusing on the ecological traits of the microbes and hosts, mediated through their shared ephemeral habitat of a rotting fruit. We first introduce the mathematical model and focus on the independent ecologies of the microorganisms and hosts on the patchy landscape. Thereafter, we assess how dispersal-related parameters, such as the bottleneck imposed by dispersal and the number of dispersing hosts, influence the enrichment of a microorganism in the associated host. Our results illustrate how a simple interaction of host and microbial ecologies can lay the foundation for the future evolution of a more specific host–microbe association.

## Model and Results

### Microbes and Hosts Cooccur in a Patchy Environment.

We consider a microbial population and a population of hosts in a landscape of M patches that decay over time. The microbial population consists of two different types, a fast-growing type and a slow-growing type whose growth rate is reduced by c with 0≤c≤1 relative to the fast type. Starting from an initially small founder population of $N_{0}$ cells, both microbial types grow within a patch until the carrying capacity K of the patch is reached. We model this microbial growth within a patch as a stochastic process. Specifically, at each time step, a random microbial individual from the local population is chosen for reproduction with a probability proportional to its growth rate. If the population size within the patch is below the maximum size K, its offspring is added to the same patch. As soon as the maximal population size is reached, microbial growth stops, thus the microorganisms stop dividing, and the final population composition of that patch is reached. Note that choosing a stochastic equivalent of logistic growth for the within-patch microbial dynamics does not change the results (*SI Appendix*, section 2). Once the carrying capacity of a patch is reached, the microbial population can only increase by dispersing to new patches. We assume that the microbes have only a very limited potential for independent dispersal, such that spread to new patches is mainly mediated by hitchhiking on multicellular, mobile organisms. In the following, we use *Drosophila* fruit flies as an example for a dispersing host, but our model applies to all host animals with similar ecologies.

The hosts develop within a patch alongside the microbes. Once they reach adulthood, the hosts leave the patch and act as dispersal vectors by inadvertently taking a sample of the local microbial population with them. The size b of this sample is assumed to be much smaller than the patch capacity K, which is likely realistic for many host taxa, such as our focus example of insects on rotting fruit patches, where a much larger number of microbes grow on the substrate than are able to be taken up by the host. We assume that this uptake of microbes is completely random, meaning that hosts will indiscriminately take up both microbial types as a by-product of developing and feeding in a microbe-rich environment (i.e., there is no active selection by the host for the slow or fast microbial type). Similarly, for both microbial types, the host animals are simply dispersal vectors, and neither of them is inherently better at surviving the host-associated dispersal stage. Consequently, the microbial population within any specific host is, on average, a direct reflection of the microbial community structure of its source patch. Thus, the relative abundances of the two microbial types in hosts from the same patch are, on average, identical to their relative abundances within the original patch.

**Fig. 1.**
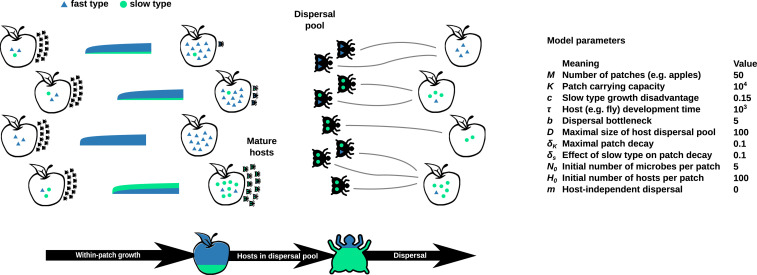
The stochastic dynamics of the slow (green dots) and fast (blue triangles) microbial types growing in a patchy landscape, illustrated by the patch-specific growth curves. Patches with a higher relative abundance of the slow type decay more slowly, allowing more hosts to develop to maturity. A random sample of the surviving mature hosts enters the dispersal pool, and each host takes a small random sample of the local microbial population with it. These hosts then lay their eggs in randomly assigned new patches, while the hitchhiking microbes provide the founding microbial population. This starts a new cycle of within-patch growth of microbes and development of hosts. The hosts in the dispersal pool are generally enriched in the slow type, because of prolonged survival of developing hosts in slower-decaying patches, setting the stage for a more specific association between the hosts and the slow type. It also allows for the survival or even gradual increase of the slow microbial type across all patches. The timeline at the bottom illustrates the consecutive phases of the dynamics and shows how, on average, the relative abundance of the slow type is higher within hosts in the dispersal pool than in the patches. The table on the right provides an overview of the model parameters and their definitions. The values given are the ones used for all simulations, unless otherwise stated. See *Model and Results* for further details.

From the population of all adult hosts on all patches, a maximum of D randomly picked hosts eventually enter a common dispersal pool. From the dispersal pool, the hosts are randomly assigned to M new patches, where each host lays $H_{0}$ eggs which found a new generation of hosts. Thus, each patch receives between zero and $n\;H_{0}$ eggs, where n is the number of hosts assigned to this patch. Note that this allows for multiple host animals to simultaneously colonize the same patch and for patches to remain uncolonized. Along with the eggs, the hosts bring in the hitchhiking microbes they have picked up at their source patches to start a new within-patch cycle of microbial population increase. The microbes introduced to the new patch increase according to the stochastic population dynamics described above. A sketch of the full model alternating between a within-patch growth cycle and a dispersal phase is shown in Fig. 1. For details of the model, see *Methods*.

### Entanglement of Host Development and Microbial Growth within Patches.

We now introduce two factors which, together, have the potential to lead to an overrepresentation of one of the microbial types within the host animals, that is, a tipping of scale toward a more specific interaction with one of the types. The first factor is that patches decay in quality over time, and this decay is detrimental to the development of hosts. There are several not mutually exclusive reasons for this patch decay. For example, patch quality for the hosts may be directly linked to the availability of resources, which decline over time as the growing microbial populations consume these resources. In this scenario, the microbes act as competitors to the developing hosts. In a related scenario, other microbes or animals may tap into the resources a patch provides, thereby leading to a gradual decline of resources up to the complete destruction of a patch. Whatever the reasons for patch decay, the survival probabilities of developing hosts decline with deteriorating patch quality. The second factor is that the slowly replicating microbial type delays the decay of a patch. This could be due to either a reduced uptake of resources as a consequence of slower growth or an active role in protecting the patch against other competitors, for example, through the costly production and release of a metabolite toxic to competitors (11, 15).

The survival of hosts depends, at least in part, on how fast the patch is decaying, which, in turn, is influenced by the abundances of the two microbial types. Here we assume that a patch decays faster with increasing absolute abundances of the fast type ($N_{f}$) and of the slow type ($N_{s}$) within the patch. Specifically, assuming that the relationship between patch decay and host survival is proportional to the sum of the microbial abundances, we write the decay probability of a host at each time step as

$$\delta =\delta_{K}\;\frac{N_{f}+\delta_{s}\;N_{s}}{K}.$$ [1]

Here $0\leq \delta_{K}\leq 1$ denotes the maximal host decay when a patch is fully occupied by the fast type (i.e., $N_{f}/K=1$), and $0\leq \delta_{s}\leq 1$ accounts for the reduced impact of the slow type on patch decay. The host decay probability Eq. **1** provides a measure of patch quality, which can be defined as $\rho =1-\delta /\delta_{K}$. Empty patches have the maximal quality ρ=1, which gradually decreases as microbial abundances (and thus δ) increase, until the minimal quality $\rho =\left(1-\delta_{s}\right)\;N_{s}/K$ is reached when a patch is fully occupied (i.e., $N_{f}+N_{s}=K$). It is important to note that, despite the reduced impact of the slow type on patch quality, Eq. **1** reflects that both microbial types have a direct detrimental effect on the development of hosts within a patch.

The ability of the microbes and hosts to colonize new patches now depends on two opposing processes with distinct timescales. The first one is the time it takes for the hosts to fully develop. Here we assume that each host takes τ time steps to maturation. The second timescale is determined by the survival time of developing hosts, that is, the average number of time steps until all hosts within a patch are dead. We derive analytical formulas for the survival of hosts in important special cases in *SI Appendix*, section 1, showing that hosts indeed, on average, survive longer in patches with higher abundances of the slow type (see *SI Appendix*, Fig. S1). The entanglement of these two timescales leads to an indirect interaction between the microbial population and the developing hosts within a single patch. If the development time τ of the hosts is longer than their survival time, on average, no host animal reaches maturation, and the hosts eventually die out. If, on the other hand, hosts mature very quickly, they are not affected by patch decay, and most hosts reach maturity regardless of the composition of the microbial population. Between these two extremes, however, the number of surviving hosts from a specific patch depends on the composition of the microbial population within that patch.

**Fig. 2.**
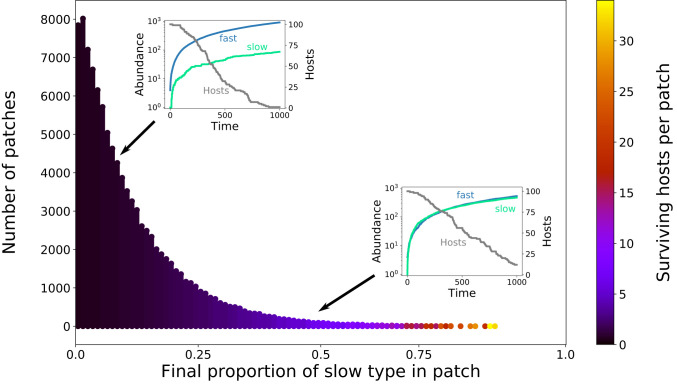
Histogram of the final proportions of the slow microbial type in 100,000 patches, each starting with an initial microbial population of one slow and four fast types, and the corresponding expected number of surviving hosts per patch (color coded). As expected, the fast type dominates in almost all patches, with the slow type mostly reaching relative abundances of less than 20%. A typical example of the corresponding within-patch population growth is shown in *Top Left Inset*. However, due to the stochastic population dynamics, in particular during the low abundances at the start of the growth cycle, in some patches, the slow type reaches relative abundances comparable to the fast type. An example of this is shown in the *Bottom Right Inset*. The coloring of the bars indicates the average number of hosts surviving in the corresponding patches. Each patch initially receives 100 developing hosts, and, in patches with a higher abundance of the slow type, which slows down patch decay, on average, a higher number of those hosts survive. Parameter values: $M=50,\;K=10^{4},\;D=100,\;c=0.15,\;H_{0}=100,\;b=5,\;\tau =10^{3},\;\delta_{K}=0.1,\;\delta_{s}=0.1$.

The composition of the microbial populations will generally vary between different patches, since population growth is a stochastic process. If the initial abundance of the fast type is low compared to the carrying capacity K, and if then a single slow type is introduced, the fast type will typically reach much higher abundances than the slow type (Fig. 2). However, despite the clear local advantage of the fast type, its relative abundance across patches can vary considerably due to stochastic effects, which are especially pronounced when population size is small. This variation then entails random differences in the survival rate of developing hosts between patches, ultimately resulting in considerable variation in the number of hosts leaving a patch (Fig. 2).

### Multiple Patches and Dispersal Can Favor the Slowly Growing Microbial Type.

**Fig. 3.**
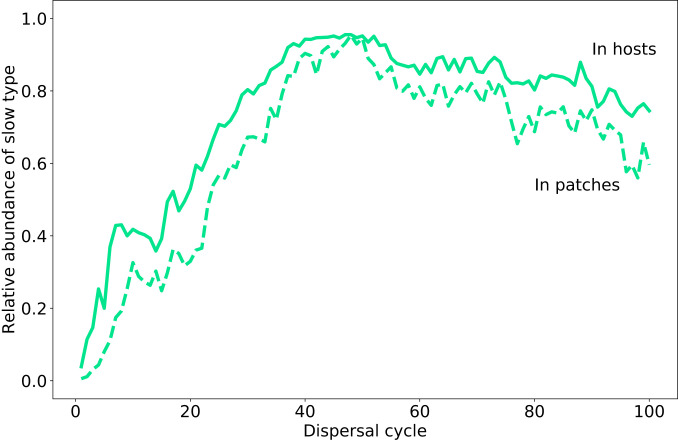
Example of the average relative abundance of the slow microbial type within patches and within hosts. The relative abundance f of the slow type during the host-associated phase is generally higher than in the patches themselves; that is, the hosts are enriched in the slow type. Parameters: $M=50,\;K=10^{4},\;D=100,\;c=0.15,\;H_{0}=100,\;b=2,\;\tau =10^{3},\delta_{K}=0.1,\;\delta_{s}=0.1$.

**Fig. 4.**
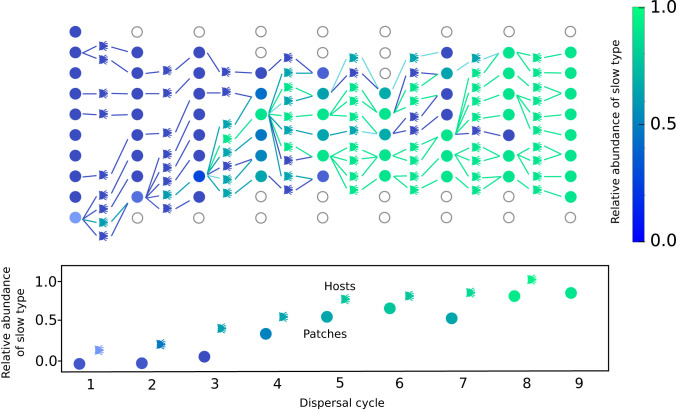
Illustration of a simulation showing the gradual increase and eventual fixation of the slow microbial type across a maximum of 10 patches after several growth cycles, despite growing slower in all patches. We start from a single slow type in one patch. Patches are colored according to the relative abundance of the slow type at the end of a growth cycle, with empty circles denoting noncolonized patches. Each patch contributes a specific number of hosts to the dispersal pool, which are also color-coded according to the relative abundance of the slow type within each individual host. The hosts are then randomly assigned to a new patch, where the growth cycle restarts. Clearly, patches with a higher abundance of the slow type contribute, on average, more hosts to the dispersal pool, which colonize more patches, thus leading to a gradual increase and eventual fixation of the slow type across all patches. The graph at the bottom illustrates both this increase of the relative abundance of the slow type across patches and its higher abundance within hosts compared to the patch environments. Parameters: $M=10,K=10^{4},\;c=0.15,\;H_{0}=100,\;b=5,\;D=10,\;\tau =10^{3},\;\delta_{K}=0.1,\;\delta_{s}=0.1$.

Even though, locally, the slow microbial type is outcompeted by the fast type, and the microbiota of hosts directly reflect the community compositions of their respective source patches, the hosts in the dispersal pool are, on average, enriched in the slow type. This is because patches with a higher abundance of the slow type decay more slowly and thus allow more hosts to mature. Thus, patches in which the slow type is more abundant, on average, contribute more hosts to the dispersal pool, which necessarily carry a higher abundance of the slow type, reflecting the population structure of their source patch. This enrichment of the slow type within dispersing hosts is a central, dynamic effect in our model, which increases the chances of a new patch being colonized by a host enriched in the slow type. This counteracts the within-patch advantage of the fast-growing microbial type and can allow the slow and fast types to coexist over many growth and dispersal cycles (Fig. 3), until, eventually, one of the two goes to fixation. This process is illustrated in Fig. 4 across a few exemplary patches and growth cycles.

**Fig. 5.**
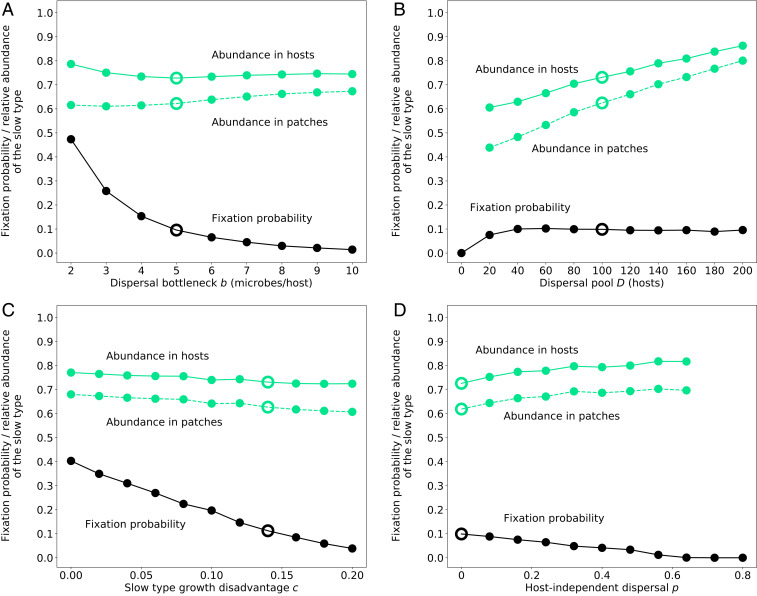
Fixation probability of a single invader of the slow microbial type and corresponding average abundance of the slow type in patches and in hosts as a function of (*A*) the size of the dispersal bottleneck imposed by hosts, (*B*) the size of the host dispersal pool, (*C*) the fitness disadvantage of the slow type, and (*D*) the number of microbes dispersing independent of hosts relative to the maximally possible number of microbes dispersing within hosts (p=m/(b D)). Importantly, the average relative abundance of the slow type is always higher in the hosts than in the patches. In all cases, each data point represents the average of 10,000 stochastic simulations. The open circles denote the parameter values used in the other panels, and the remaining fixed parameters are $M=50,\;K=10^{4},\;H_{0}=100,\;\tau =10^{3},\;\delta_{K}=0.1,\;\delta_{s}=0.1$.

Since each host picks up only a very few microbes compared to the total local population, that is, b≪K, the dispersal stage poses a bottleneck for the microbes. The fixation of the slow type becomes more likely when this dispersal-induced bottleneck becomes more severe (Fig. 5*A*). This is because, by chance, some hosts will pick up only slow-growing cells and initiate, after dispersal, pure slow-type patches. Such pure slow-type patches allow a disproportionate number of hosts to mature, all of which then again carry only slow microbial types to new patches. As a consequence, a pure slow-type lineage can rapidly expand and cause displacement of less advantageous, in terms of host survival, fast-type or mixed patches. If the bottleneck is small enough, this can then lead to the extinction of the fast microbial type and the fixation of the slow type in all patches (Fig. 5*A*). The size of the dispersal pool, that is, how many hosts disperse compared to the number of available patches, has a relatively small effect on the slow type’s fixation probability (Fig. 5*B*). The fixation probability initially increases slightly, but, as soon as each new patch receives, on average, at least one host, it remains largely unaffected by a further increase in the number of host animals in the dispersal pool.

As expected, fixation of the slow type becomes less likely the greater its fitness disadvantage relative to the fast type (Fig. 5*C*). In general, the impact of the slow type on patch decay is independent of its fitness, and, for a fixed growth rate of the slow type, its fixation is most likely when it does not contribute to patch decay at all (see *SI Appendix*, Fig. S3*A*). The more the slow type contributes to patch decay, the fewer hosts survive in the patch and the less likely becomes the slow type’s survival and fixation. When it has essentially the same impact as the fast type (i.e., $\delta_{s}$ close to one), survival of the slow type becomes virtually impossible (see *SI Appendix*, Fig. S3*A*). An interesting scenario arises when the slowly growing type reduces patch decay through reduced uptake of resources. In this case, the fitness of the slow type and its contribution to patch decay are coupled. If the slow type’s contribution to patch decay decreases with increasingly disadvantageous fitness relative to the fast type, there is an optimal growth rate of the slow type at which survival and fixation are most likely (see *SI Appendix*, Fig. S3*B*). This optimal growth rate emerges at an intermediate value, where the slow type’s fitness is still high enough to allow it to occasionally reach high abundances within a patch, but not so high that it leads to rapid patch decay. This represents a balance between within-patch competition (high growth rate) and dispersal (low impact on host development).

This analysis reveals the crucial role of microbial growth rate and patch decay in shaping microbial abundance in the host. To further test whether the results depend on the specific within-patch growth dynamics as described above, we replaced the corresponding stochastic process with a stochastic equivalent of logistic growth (*SI Appendix*, section 2). This yields almost identical results (*SI Appendix*, Figs. S5 and S6), showing that the effect of enrichment of the slow type does not critically depend on the specific form of microbial growth.

In all these scenarios, we have assumed that host-mediated dispersal is the only route by which microbes can reach new patches. It is, however, possible to relax this assumption and allow for host-independent microbial dispersal between patches. We include such host-independent dispersal by sampling m microbes from across all patches at the time of host dispersal, and assigning them randomly to new patches. This process is similar to assuming that patches already contain small resident populations of microbes. Because the sampling from the source patches is random, the compositions of these initial populations, on average, directly reflect the composition of the metapopulation of microbes in the source patches. This type of global dispersal thus leads to increased nonassortative mixing of the microbial populations on the patchy landscape, which generally favors fast growth. But, over a large range of the relative sizes of the host-independent microbial dispersal pool, the previous results still hold (Fig. 5*D*). Only when host-independent dispersal between patches approaches levels similar to host-mediated dispersal will the fast-growing type be almost universally favored. This shows that a patchy environment and dispersal limitation is indeed crucial for the observed results.

### Enrichment of the Slow Type in the Host.

The observed survival and fixation of the slow type is possible, despite its fitness disadvantage, because the relative abundances of the slow microbial type are always higher in the dispersing hosts than in the patches (Fig. 5). This is a key result of our model, which does not depend on the slow type being specifically beneficial to the host or being actively picked up by it. We explicitly assumed that, just like the fast microbial type, the slow type impairs development of hosts within a patch. But, crucially, it does so to a lesser extent than the fast type. The enrichment of the slow type is thus a consequence of patches with higher abundances of the slow type disproportionately contributing to the host dispersal pool, rather than a specific beneficial effect of the slow type.

This association between the slow type and the hosts is not only a general pattern; the host metapopulation is also very stable against reinvasion of the fast microbial type. This is because, if a patch is invaded by a fast-growing type, this particular patch will not allow many hosts to mature, while the remaining pure slow-type patches contribute a very high number of hosts to the dispersal pool. So, even if a few hosts survive in the invaded patch, the resulting mature hosts carrying fast-growing types are diluted out in the dispersal pool and only rarely colonize new patches. This makes dispersal from an invaded/contaminated patch to new patches less likely, thus effectively containing invading fast types in a self-imposed quarantine.

### Sterile Patches.

So far, we have assumed that, initially, all patches are occupied by microbes. Under these conditions, hosts can never develop in a completely microbe-free (or sterile) patch, since they will always bring a founding microbial population from their nonsterile source patch. But Eq. **1** implies that hosts would, in fact, perform best on sterile patches, as hosts do not decay at all under those conditions (i.e., δ=0). To investigate whether our results still hold when hosts can develop in sterile patches, we repeated our simulations with only 10% of the patches initially being colonized by microbes. In this case, the sterile patches initially contribute the vast majority of hosts to the dispersal pool, since there is no decay of hosts in these patches. In the absence of host-independent dispersal, this means that the microbes die out within the first few dispersal cycles, resulting in almost all patches and all hosts remaining sterile afterward (see *SI Appendix*, Fig. S4). However, with increasing host-independent dispersal, complete sterility of patches, and hosts, becomes harder and harder to maintain. Then, as in our previous results, patches with a higher proportion of the slow type contribute more hosts to the dispersal pool, making the spread of the slow type and its fixation more likely (see *SI Appendix*, Fig. S4). In contrast to the case when all patches were initially occupied by microbes (Fig. 5*D*), increasing host-independent dispersal thus increases the fixation probability of the slow type when the majority of patches are initially sterile. In fact, in this scenario, small to intermediate amounts of dispersal independent of hosts make it even more likely for the slow type to go to fixation, as it encounters reduced competition from the fast type within the metapopulation across all patches. But, as before, when host-independent dispersal becomes the dominant mode of dispersal, its mixing effect favors fast growth and leads to a decline of the fixation probability of the slow type (see *SI Appendix*, Fig. S4). When many patches are initially microbe free, an intermediate level of host-independent dispersal is thus optimal for the slow-growing type and its association with the host. In general, sterile patches, and thus microbe-free hosts, can only be maintained under the restrictive assumption that sterile patches exist initially and that there is only very little host-independent dispersal. In more realistic scenarios, microbial populations will occupy the majority of patches, making it unlikely for the hosts to avoid codevelopment with microbes.

### Timing of Host Dispersal.

In our model, we have assumed that all surviving hosts across all patches disperse synchronously in evenly spaced intervals determined by the host development time τ. At the same time, a completely new set of patches is available for the dispersing hosts to colonize. Our analysis of the survival of hosts within patches over time (*SI Appendix*, section 1 and Fig. S1) indicates that this assumption can be relaxed, as there is, in fact, a time window for host dispersal which is optimal with respect to enrichment of the slow type. If hosts develop quickly and disperse early, they are not affected by patch decay and enrichment, and fixation of the slow type is unlikely (*SI Appendix*, Fig. S2). Conversely, if the development time is too long, the hosts do not survive until dispersal and fixation of the slow type is again unlikely (*SI Appendix*, Fig. S2). This shows that enrichment of the slow type is most likely if hosts disperse in a time window that is mainly determined by the decay rate given in Eq. **1**.

But this effect of host dispersal timing does not require that all hosts disperse synchronously at the same time, as we will now show. We consider a generalized version of the model where each individual host has a random development time drawn from a uniform distribution on the interval $\left[\tau -\tau_{d},\tau +\tau_{d}\right]$ around a mean development time τ. We further generalize the model by relaxing the assumption that there is a complete turnover of patches coinciding with host dispersal. We do this by replacing a randomly sampled number $M_{0}\leq M$ of patches with empty patches at random times $t_{i}$. The synchronous model we have discussed above then corresponds to the special case $\tau_{d}=0$ and $t_{i}=\tau$ for all i with complete patch renewal (i.e., $M_{0}=M$).

We illustrate the dynamics of this more general model by letting the development times of individual hosts vary by $\tau_{d}=200$ around a mean value of $\tau =10^{3}$. These hosts now migrate on a landscape of M=100 patches, of which a single patch ($M_{0}=1$) is renewed at, on average, every 100 time steps. As a consequence, the synchronized cycles of dispersal and within-patch microbial growth across all patches are broken, and the majority of patches are occupied by growing microbial populations at all times (*SI Appendix*, Fig. S7*A*). The corresponding dynamics in the host metapopulation show fluctuations around long-term averages, with marked differences depending on whether the slow type can spread across the patches or not (*SI Appendix*, Fig. S7*B*). While the hosts can survive on pure fast-type patches, the average number of live hosts per patch is much higher when the slow type is able to spread (*SI Appendix*, Fig. S7*B*). In line with our above results for synchronous host dispersal, when the slow type spreads across the patches, its average relative abundance is always higher in dispersing hosts compared to its relative abundance within the patches (*SI Appendix*, Fig. S7*C*).

## Discussion

The central result of our analysis is that one of the microbial types becomes enriched in the host population without any prior evolutionary adaptation to exploiting the host or any effect on host fecundity, the number of patches visited by the hosts, or preference for slow-growing microbes by the dispersing hosts. Instead, we observed a shift of the microbial community in the patches and a significant enrichment of the slow microbial type in the host, only because microbial and host life cycles are interconnected. This shift was observed, even though the slow-growing microbes are competitively inferior in the individual patches in comparison to the fast-growing microbes. The most important reasons are that 1) microbes disperse via the host, 2) microbes change habitat conditions in a way that affects host development, and 3) the microbe bottleneck size during dispersal can favor initially rare types. These interconnections between microbial and host life cycles may not be restricted to our reference example, consisting of rotting fruits and insects as dispersal vectors for the microbes. Similar shifts and similar host-associated enrichment of microbes are expected whenever there are opportunities for microbes to disperse via hosts in patchy habitat environments and/or when microbes slow down habitat deterioration for the host. Such conditions occur for any ephemeral habitat type, including seasonal plants (which serve as temporary habitat for diverse invertebrates) or temporary ponds. It may also apply to eukaryotic parasites or phytophagous insects, which inhabit other hosts and could benefit from microbe-mediated changes of their host environment or enhance microbe dispersal. There are also similarities with the evolution of complex life cycles in parasites, in particular, the incorporation of hosts on higher trophic levels, which have been suggested to primarily enhance parasite dispersal (16, 17).

Our reference example is inspired by the *Drosophila* habitat of microbe-infested, rotting fruits, yet the described conditions are not intended as an accurate representation of the *Drosophila* interactions with its orally acquired gut microbiota. Indeed, there is evidence that specific members of the gut microbiota can promote both the developmental rate and fecundity of *Drosophila* and affect its locomotory activity (14, 18, 19), and that *Drosophila* can utilize microbial volatile cues in its feeding choices (20). Nevertheless, our results provide a candidate scenario for the evolutionary origins of these associations in fruit flies and other hosts in ephemeral habitats. Specifically, ecological fit between the life history traits of slow-growing microorganisms and an animal dispersal agent utilizing a resource patch can promote cooccurrence. Because of their overlapping selective interests, this cooccurrence can lead to adaptations by both partners for greater coordination of life cycles and increased codispersal. The importance of partner fidelity in the evolution of animal–microbial symbioses has been considered widely, but primarily in the context of vertical transmission as a selective force that ameliorates deleterious traits in the microbial partner (6, 21, 22). This study demonstrates the ecological conditions that favor the first candidate steps in the evolution of intimate mutually beneficial associations from interactions between animals and free-living populations of nonpathogenic microorganisms.

How widespread are symbioses that may have evolved from the cooccurrence of animals and microbes in ephemeral patches? Candidate habitats in the terrestrial environment include fleshy fruits and seeds of plants, as well as animal cadavers. These various habitats are exploited by microorganisms and insects that, as for *Drosophila*, have larval stages within the habitat patch and an adult dispersal stage. For some insect groups, the associations with microorganisms are poorly studied and apparently casual (e.g., fruit-feeding tortricid moths, seed-infesting bruchid beetles), but other associations are highly specific, and characterized by microbial services that enhance preadult insect fitness and microbial dispersal via the adult insect. For example, the bacterium *Candidatus Erwinia* dacicola protects larvae of the olive fruit fly *Bactrocera oleae* from toxic secondary compounds in unripe olive fruits (23); the yeast symbiont *Yarrowia* of burying beetles (family Silphidae) plays an essential role in preserving vertebrate cadavers inhabited by the beetle larvae (24); and larval beewolves (Hymenoptera of family Crabonidae) which feed on maternally provisioned bee prey are protected by *Streptomyces* symbionts against fungal infestation (25). Various nematodes, including *Caenorhabditis elegans*, similarly inhabit short-lived habitats with a patchy distribution, such as decomposing plant matter and rotting fruits (26). These nematodes thus need to migrate to new habitat patches at regular intervals, most likely accompanied by various microbes which readily colonize the worm’s gut in its natural environment (27). Comparable relationships have likely evolved between microorganisms and other mobile animals. Notably, the entomopathogenic bacteria *Photorhabdus* and *Xenorhabdus* are dispersed between insect hosts via juvenile nematodes (Steinernematidae, Heterorhabditidae) and enable several generations of nematode proliferation in the insect cadaver via microbicides that suppress colonization of the insect cadaver by competing bacteria (28). Our model formulations are potentially applicable to these various associations. Specifically, they suggest possible ecological conditions that favor the interactions between animals and free-living populations of nonpathogenic microorganisms that represent the first steps in the evolution of intimate mutually beneficial associations.

Our study further contributes to understanding of the ecology of fleshy fruits and other nutrient-rich ephemeral patches. Janzen (11) has argued cogently that the fate of a fruit is driven by conflict between microbe-mediated decay and the mutualism between the fruit-bearing plant and frugivores that mediate seed dispersal. Fruit-infesting insects (or other invertebrates) have long been recognized as a fourth set of players, usually treated as an antagonist of the plant–frugivore mutualism (29). Our modeling points to the possibility of a more nuanced relationship among the four players. Under conditions where fast- and slow-growing microorganisms are competing for the fruit resources, infestation by insects that disperse fruit-associated microorganisms tends to favor slow-growing microorganisms. In this way, the insects may reduce the rates of microbial-mediated fruit spoilage, ameliorating the negative consequences for the plant–frugivore partnership. Consistent with this scenario, some fermentation products of fruit-associated microorganisms promote the detection and consumption of fruits by mammalian frugivores (30, 31). The amelioration of the antagonism is not, however, symmetrical. Frugivory by a mammal or bird is generally lethal for fruit-inhabiting insects, and it is also disadvantageous for the microorganisms, because frugivore-mediated dispersal of microorganisms is less targeted to a suitable microhabitat (e.g., another fruit) than dispersal by fruit-associated insects.

Conceptually, microbial communities distributed across patches provide an example of multilevel selection (32). A recent study used such multilevel selection theory to study the evolution of host–microbe associations (33), but this model assumes that there is a direct fitness benefit of carrying one of the microbial types and that the association is already established; that is, a host-independent life stage of the microbes does not exist. This type of model can thus not capture how this association arises from free-living microbes that do not interact directly with the host. Another interpretation of the multilevel selection imposed by patchily distributed habitats and dispersal is that it acts as a scaffold conferring Darwinian-like properties on the collectives inhabiting such landscapes (34). In our case, the collectives are formed by the microbial communities inhabiting different patches, which are transmitted by host dispersal. Selection at this level should subsequently favor specificity of the microbe–host association, because specificity increases transmission and thus evolutionary success of the community.

More generally, our model of isolated patches connected by dispersal is based on some of the same principles that have been invoked as a possible route for the evolution of altruistic traits, in particular, the classical haystack model (35). The spread of the slow type can be understood as the result of assortment of microbial types during colonization of new patches, mediated by dispersal of microbes via hosts, which tends to preserve community structure and makes newly colonized patches look similar to their source patches. Consequently, some of our findings regarding the spread of the slow type are in line with well-established results from this framework (36, 37). This includes the observation that smaller bottlenecks favor the slow type (Fig. 5*A*) and the detrimental effect of host-independent, globally mixing migration between patches (Fig. 5*D*). In contrast to these studies, we are, however, not interested in the spread and maintenance of a certain (e.g., slow) type per se, but in its enrichment in the hosts relative to within-patch communities. In our model, communities are imperfectly copied by dispersing hosts, and we lay the focus on the effect of these communities on the success of the dispersal agent. This success then feeds back into differential spread and survival of the microbial types across the patches. This mode of migration, namely, a dispersal agent carrying a fixed number of individuals to a new patch, is conceptually similar to the propagule migration discussed in ref. 38. Multilevel selection and haystack models, however, put the emphasis on the patches and mostly neglect the details of how groups or communities expand in the population, while studies focusing on dispersal of species on patchy landscapes usually assume fixed dispersal rates (39). The interplay of dispersal and more prudent resource use also has important parallels to the dynamics of host–pathogen interactions, for which it was shown that an externally imposed, restricted migration pattern favors the evolution of more prudent pathogens, counteracting the risk of local extinctions (40). In contrast, unrestricted migration favors more-virulent, or rapacious, pathogens because of continued access to new hosts. Our model is distinct from this example of host–pathogen evolution in that we do not impose and fix the level of dispersal, allowing for a dynamic feedback between the relative abundances of the microbial types and the rate of dispersal.

Over evolutionary timescales, we would expect both host and slow-growing microbe to evolve a closer, more specific association. The hosts could, for example, actively seek out patches with a high abundance of the slow type, potentially following cues provided by the slow type. Moreover, evolutionary changes in the host immune system may promote the specific uptake of the less detrimental microbe and/or a generally reduced uptake of microbes, thereby enhancing the quality of the subsequently colonized patches. The microbes could also evolve traits that enhance their transport by dispersing hosts, for example, by modulating host feeding behavior (41), and improve patch quality by suppressing growth of competing microbes (e.g., those of the fast type). These examples are subsequent steps to a mutual microbe–host interaction, which could (but need not) be favored by selection as a consequence of the established association that simply follows from the overlap of lifestyles.

Our results call for caution in explaining the causes for overrepresented microbial taxa in host-associated microbial communities. The observed enrichment of the slow microbial type in adult hosts may be (mis-)taken as an indication that this microbe enhances host fitness. This is, however, not the case; the slow type increases neither the number of an individual host’s offspring nor the number of patches a dispersing host visits. On the contrary, in our model, fitness would be maximized for hosts developing in a sterile environment. To better understand why the slow type is enriched in the hosts, it is instructive to focus on the scenario where microbes and developing hosts compete for resources within a patch. If the slow type consumes those resources at a slower rate than the fast type, then, from the host’s perspective, it is the lesser of two evils, as this will prolong resource availability and thereby extend the time window for host maturation. In this scenario, the persistent enrichment of the slow type in the hosts is purely a collateral effect of ecological competition within the patches. As we have shown, this is entirely an effect of the entanglement of an aspect of the free-living stage of the microbe, the host life cycle, and the host-associated dispersal across distinct patches. The dispersal stage is crucial, as it represents a bottleneck which accentuates the patch-to-patch variation in the abundance of the slow type and thus exposes it to increased selection. If any one of these aspects of the ecological context is removed, for example, by hosts developing in a continuous environment without patches and dispersal, enrichment of the slow type would not be observed. In particular, we observed that a substantial number of initially sterile patches and limited microbial dispersal makes survival, and enrichment, of microbes unlikely. While this scenario may rarely be encountered in natural settings, it nevertheless provides an additional prediction of which conditions should be expected to favor the enrichment of certain microbes, and which conditions should not.

In summary, we have shown how the interplay between initially unrelated aspects of the ecology of microorganisms and an animal host can lead to a closer host–microbe association, without specific beneficial effects or choice by either of the interaction partners. Our analysis provides the basis for specific hypotheses that can be tested empirically, to investigate the often-assumed adaptive origin of prevalent host–microbe associations.

## Methods

### Microbial Within-Patch Dynamics.

We consider a population of $N_{f}$ fast types and $N_{s}$ slow types in a patch of maximal size K. At each time step, the probabilities for the abundance of the fast ($N_{f}$) or the slow ($N_{s}$) type to increase by one are given by

$$\begin{array}{cc} P\left(N_{f}\to N_{f}+1\right) & =\frac{N_{f}}{N_{f}+\left(1-c\right)\;N_{s}}\\ P\left(N_{s}\to N_{s}+1\right) & =\frac{\left(1-c\right)\;N_{s}}{N_{f}+\left(1-c\right)\;N_{s}}, \end{array}$$ [2]

for $N_{f}+N_{s}<K$, and $P\left(N_{f}\to N_{f}+1\right)=P\left(N_{s}\to N_{s}+1\right)=0$ otherwise. The factor 0≤c≤1 accounts for the reduced growth rate of the slow type. This defines a pure birth process without death; that is, in each time step, the population increases by one and never decreases.

### Host Decay and Dispersal.

Denote by H the number of live hosts at a certain time within a patch with microbial abundances of $N_{f}$ fast types and $N_{s}$ slow types. The probability of each host to die is given by Eq. **1**, and the total number $H_{\delta }$ of hosts to die at this time step is then drawn from a binomial distribution $H_{\delta }\approx B\left(H,\delta \right)$ with mean H δ.

After τ time steps for each surviving host, a sample of size b is randomly drawn without replacement from the local microbial population. Since b is constant and independent of the current size of the microbial population, uptake is modeled in terms of the proportions of the microbial types, not their absolute abundances. This is reasonable because the numbers taken up by an individual host are orders of magnitude lower than the total pool available in the patch. Moreover, we assume that the number of hosts will only ever decrease in a patch, so that, even if microbes grow extremely slowly, the number of microbes dispersing with hosts is still small compared to the total number of microbes within a patch. The microbial samples are then randomly assigned to M new patches as the initial microbial populations.

## Supplementary Material

Supplementary File

## Data Availability

A Python implementation of the model underlying the results in this paper has been deposited in GitHub (https://github.com/misieber/patchbiota).
