# Supplementary material for: On the evolutionary origins of host–microbe associations
Source: Proc Natl Acad Sci U S A. 2021 Feb 22;118(9):e2016487118. doi: 10.1073/pnas.2016487118 (PMC7936343; doi:10.1073/pnas.2016487118)
Supplement: Supplementary File [file pnas.2016487118.sapp.pdf]

On the evolutionary origins of host-microbe associations
Supporting Information

1 Host survival within patches

Let $N_{f,t}$ and $N_{s,t}$ denote the abundances of the fast and slow type, respectively, and n_t the number of developing hosts in the patch at timestep t. Each host dies with probability δ_t, given by Eq. 1, and thus the total number of hosts that survives until the next timestep is binomially distributed with mean

$$n_{t+1} = n_t\,(1-\delta_t) = n_t\,\left(1-\delta_K\,\frac{N_{f,t}+\delta_s N_{s,t}}{K}\right). \tag{S1}$$

Surviving until time t requires not having died at all previous timesteps and so the average number of hosts that is still alive at time t is given by the recursion

$$\begin{aligned} n_t &= n_{t-1}\,(1-\delta_{t-1}) = [n_{t-2}\,(1-\delta_{t-2})]\,(1-\delta_{t-1}) = \dots \\ &= n_0 \prod_{i=0}^{t} (1-\delta_i) = n_0 \prod_{i=0}^{t} \left(1-\delta_K\,\frac{N_{f,i}+\delta_s N_{f,i}}{K}\right), \end{aligned} \tag{S2}$$

where n_0 is the initial number of hosts in a patch. Generally, the number of survivors at a time t depends on the specific realized stochastic microbial composition up to that time. We can look at some instructive special cases for which the survival probability depends only on time.

At every time step one microbe is added to the patch by reproduction, and there is no microbial death. In this pure birth process microbial abundance increases by one at each time step, either by birth of a fast or slow type microbe, respectively. If we start with a single microbe in an empty patch this implies that there is a direct correspondence between total microbial abundance and time, i.e. $N_{f,t}+N_{s,t}=t$ as long as $t \leq K$ (and $N_{f,t}+N_{s,t}=K$ otherwise). With this and denoting by $\eta_t = N_{s,t}/(N_{f,t}+N_{s,t}) = N_{s,t}/t$ the fraction of the slow type within a patch, the number of surviving hosts (S2) can be rewritten as

$$n_t = n_0 \prod_{i=0}^{t} \left(1-\delta_K\,i\,\frac{1-(1-\delta_s)\,\eta_i}{K}\right). \tag{S3}$$

This shows that if the slow type makes up a constant fraction of the population at all times, i.e. $\eta_i = \eta$ for all i, the survival probability only depends on time.

In Fig. S1 the number of surviving hosts n_t over time is shown for a high patch decay rate δ_K (Fig. S1a) and a low patch decay rate (Fig. S1b) and for three different constant fractions of the slow type within a patch: no slow type ($\eta = 0$), 50% slow type ($\eta = 0.5$), and only slow type ($\eta = 1$). In all cases the number of surviving hosts declines over time since both microbial types negatively affect host survival. As expected, higher proportions of the slow type within the patch results in prolonged survival of hosts. Note that a lower patch decay rate allows for an overall longer survival of hosts.

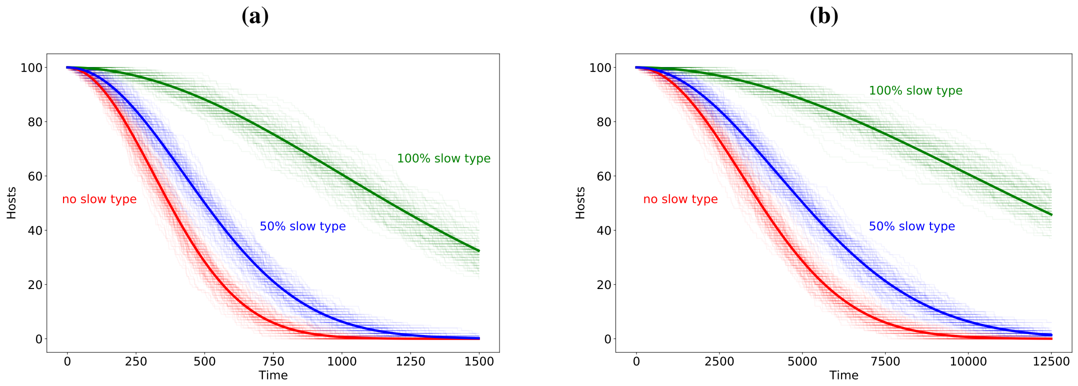

Figure S1 Number of surviving hosts over time for patches with different, fixed proportions of the slow type within the microbial population. For each case, the solid lines were obtained from Eq. S3 and the thin lines show the number of surviving hosts in 100 stochastic simulations of the model. (a) High patch decay rate $\delta_K = 0.1$. (b) Low patch decay rate $\delta_K = 10^{-3}$. Note the difference in timescale. Other parameters $M = 50$, $K = 10^4$, $c = 0.15$, $H_0 = 100$, $\delta_s = 0.1$.

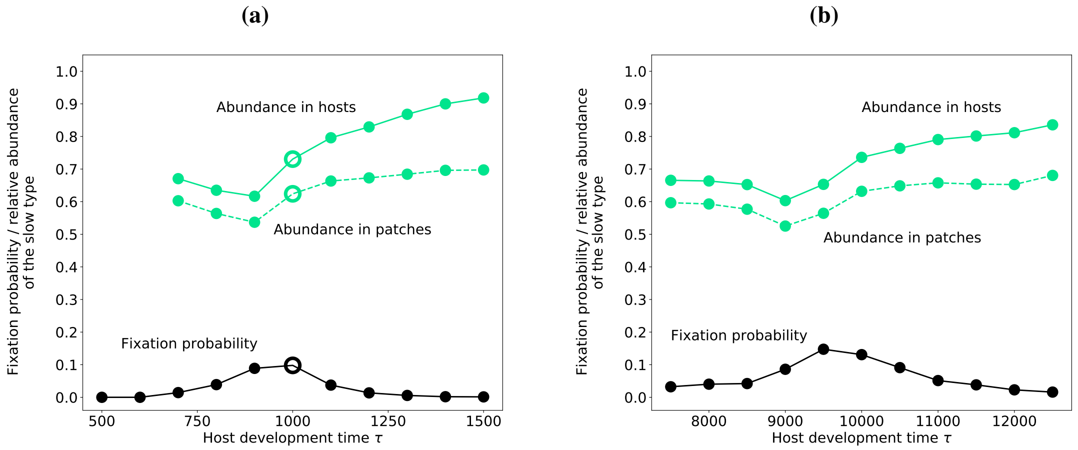

Figure S2 Fixation probability of a single invader of the slow microbial type and corresponding average abundance of the slow type in patches and in hosts as a function of host development time. (a) High patch decay rate $\delta_K = 0.1$. (b) Low patch decay rate $\delta_K = 10^{-3}$. Note the difference in timescale. The average relative abundance of the slow type is always higher in the hosts than in the patches. Each datapoint represents the average of 10000 stochastic simulations. The open circles in panel (a) denote the dispersal time used in the main text and the other fixed parameters are $M = 50$, $K = 10^4$, $b = 5$, $D = 100$, $c = 0.15$, $H_0 = 100$, $\delta_s = 0.1$

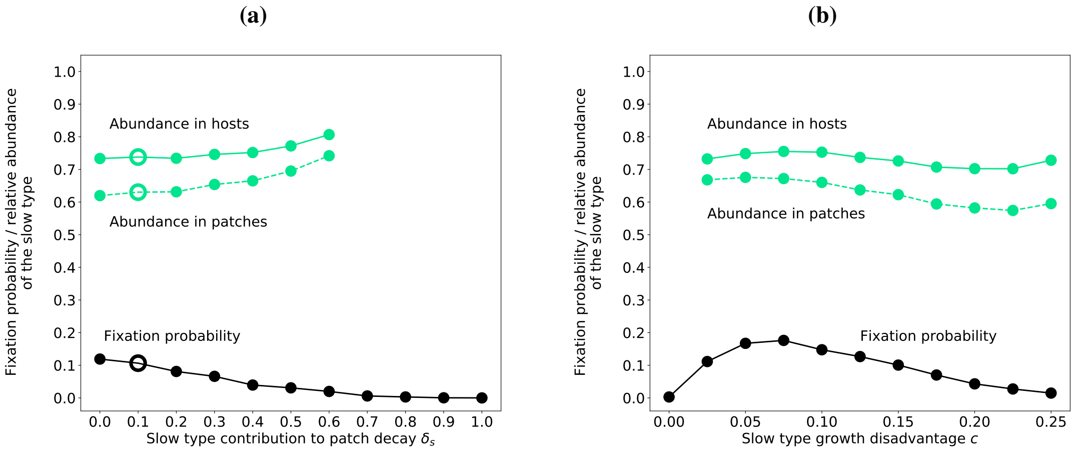

Figure S3 Fixation probability of a single invader of the slow microbial type and corresponding average abundance of the slow type in patches and in hosts as a function of (a) the impact of the slow type on patch decay when its impact on patch decay is independent of its growth rate disadvantage, which is fixed at $c = 0.15$, (b) the growth disadvantage of the slow type when its impact on patch decay decreases with increasing disadvantage (here for example $\delta_s = \exp(-kc)$ with $k = 15$). The average relative abundance of the slow type is always higher in the hosts than in the patches. In all cases, each datapoint represents the average of 10000 stochastic simulations. The open circles in panel (a) denote the parameter value used in the main text and the other fixed parameters are $M = 50$, $K = 10^4$, $b = 5$, $D = 100$, $H_0 = 100$, $\tau = 10^3$, $\delta_K = 0.1$

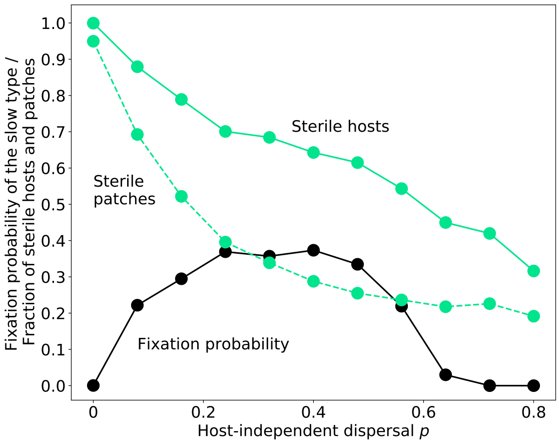

Figure S4 Fixation probability of a single invader of the slow microbial type and fraction of patches and hosts that remain uncolonized by microbes as a function of the number of microbes dispersing independent of hosts relative to the maximally possible number of microbes dispersing within hosts ($p = m/(bD)$). Here, initally only 10% of the patches are colonized by microbial populations. Each datapoint represents the average of 10000 stochastic simulations and the fixed parameters are $M = 50$, $K = 10^4$, $b = 5$, $D = 100$, $c = 0.15$, $H_0 = 100$, $\tau = 10^3$, $\delta_K = 0.1$, $\delta_s = 0.1$

2 Logistic microbial growth

For the model with a logistic-like growth dynamics we replace the transition probabilities given by Eq. 2 with

$$P(N_f \to N_f + 1) = \frac{N_f}{K}\left(1 - \frac{N_f + N_s}{K}\right)$$
$$P(N_s \to N_s + 1) = \frac{(1-c)N_s}{K}\left(1 - \frac{N_f + N_s}{K}\right),$$

As before, the factor $1-c$ with $0 \le c \le 1$ accounts for the reduced growth rate of the slow type. This also defines a pure birth process without death, i.e. in each timestep the population increases by one and never decreases. All other aspects of the model, such as host decay and dispersal, remain unchanged.

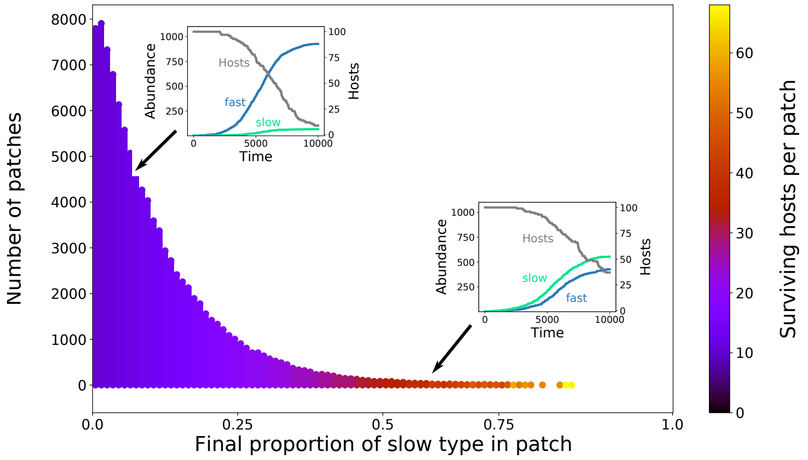

Figure S5 Histogram of the final proportions of the slow microbial type in 100,000 patches with logistic microbial growth dynamics, each starting with an initial microbial population of 1 slow and 4 fast types, and the corresponding expected number of surviving hosts per patch (color coded). As expected, the fast type dominates in almost all patches, with the slow type mostly reaching relative abundances of less than 20%. A typical example of the corresponding within-patch population growth is shown in the top-left inset. However, due to the stochastic population dynamics, in particular during the low abundances at the start of the growth cycle, in some patches the slow type reaches relative abundances comparable to the fast type. An example of this is shown in the inset at the lower right. The coloring of the bars indicates the average number of hosts surviving in the corresponding patches. Each patch initially receives 100 developing hosts and in patches with a higher abundance of the slow type, which slows down patch decay, on average a higher number of those hosts survives. Parameter values: $M = 50$, $K = 10^4$, $c = 0.15$, $H_0 = 100$, $b = 5$, $\tau = 10^3$, $\delta_K = 0.1$, $\delta_s = 0.1$.

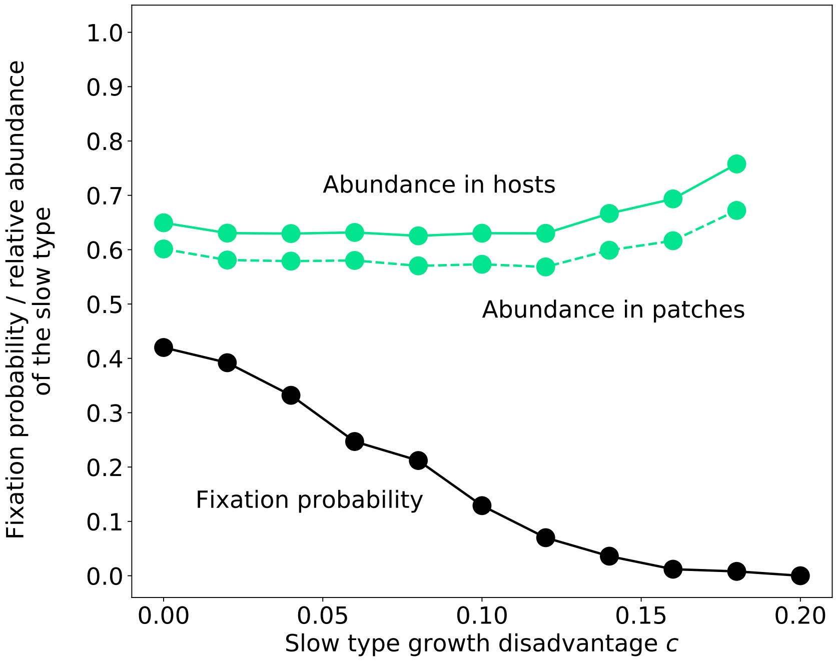

Figure S6 Fixation probability of a single invader of the slow microbial type and corresponding average abundance of the slow type in patches and in hosts as a function of the slow type's growth disadvantage for the model with logistic microbial growth dynamics. The average relative abundance of the slow type is always higher in the hosts than in the patches. Each datapoint represents the average of 10000 stochastic simulations and the fixed parameters are $M = 50$, $K = 10^4$, $b = 5$, $D = 100$, $c = 0.15$, $H_0 = 100$, $\tau = 10^3$, $\delta_K = 0.1$, $\delta_s = 0.1$

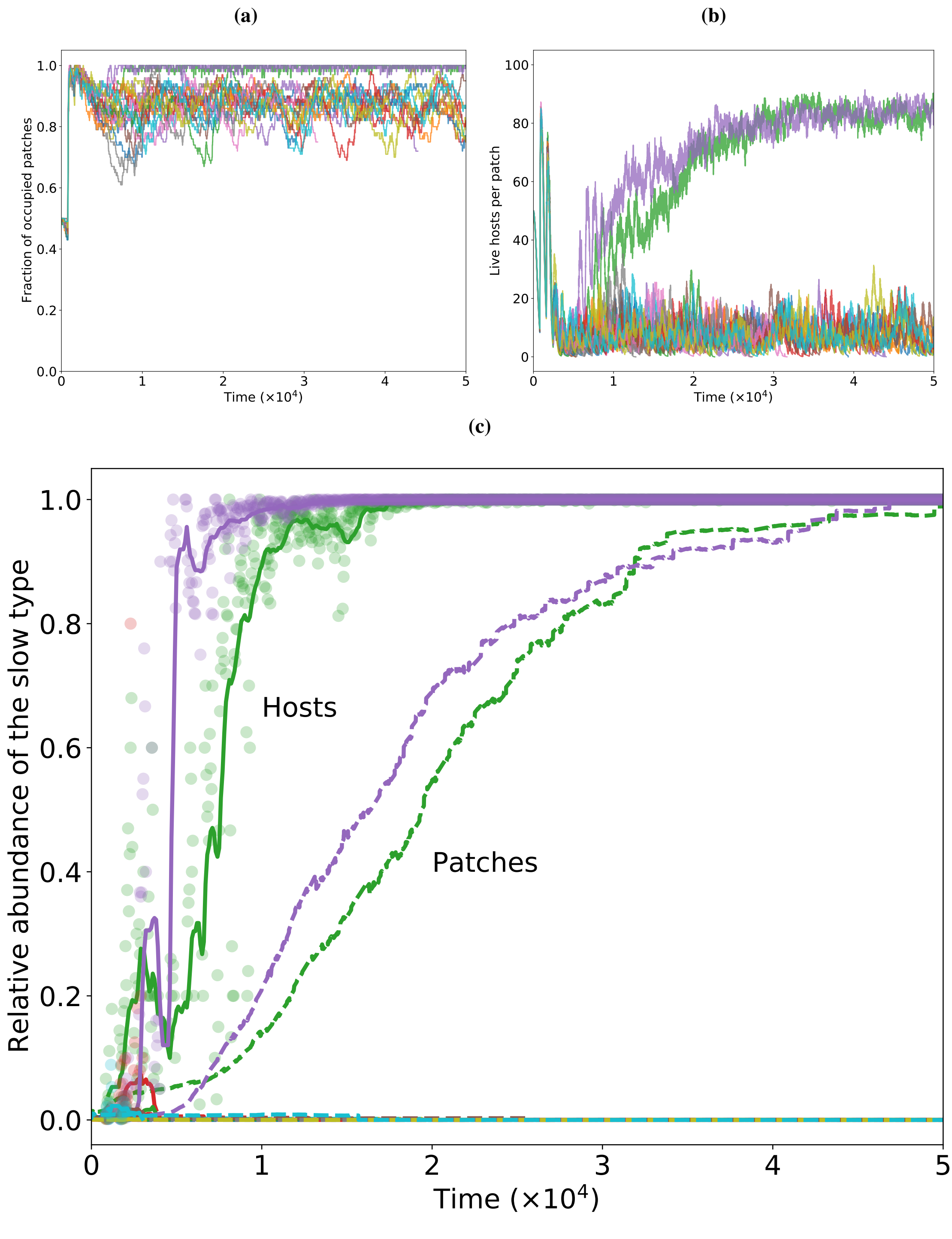

Figure S7 Ten stochastic realizations of the model with asynchronous host dispersal. Different colors denote different realizations and colors are consistent across all panels. (a) The fraction of patches occupied by microbes over time. The two realizations where the slow type could spread have almost 100% patch occupancy at all times, while about 10% of patches remain unoccupied when only the fast type spreads. (b) The average number of surviving hosts per patch over time. When the slow type can spread across the patches more hosts survive per patch. (c) Mean relative abundance of the slow type in patches and hosts over time. The line for the hosts shows the moving average over 1000 timesteps and the points illustrate the population composition within individual hosts at their respective dispersal times. In two realizations the slow type increases in abundance and its abundance is higher in dispersing hosts than in the patches. The parameters of the asynchronous model are $M = 100$, $M_0 = 1$, $K = 10^4$, $b = 5$, $c = 0.15$, $H_0 = 100$, $\tau = 10^3$, $\tau_d = 200$, $\delta_K = 0.1$, $\delta_s = 0.1$
